# Haploidentical donor versus double-unit cord blood transplantation in hematologic malignancies: comparative survival and graft-versus-host disease outcomes from a decade of real-world experience

**DOI:** 10.1186/s12885-026-16221-w

**Published:** 2026-05-22

**Authors:** Rui Liu, Jie Zhao, Xiaohui Deng, Yan Jiang, Siguo Hao, Jiangbo Wan

**Affiliations:** Department of Hematology, Xinhua Hospital, Shanghai Jiaotong University School of Medicine, 1665 Kongjiang Road, Shanghai, 200090 China

**Keywords:** Haploidentical donor, Double umbilical cord blood, Hematopoietic stem cell transplantation, Graft-versus-host disease, Engraftment, Survival outcomes, Alternative donor transplantation

## Abstract

**Background:**

Allogeneic hematopoietic stem cell transplantation (HSCT) is a curative option for patients with hematologic malignancies, but the limited availability of HLA-matched sibling donors necessitates alternative graft sources. Haploidentical donors (HIDs) and double-unit umbilical cord blood (DUCB) are two widely used alternatives, yet direct comparisons of their clinical outcomes remain limited.

**Methods:**

We conducted a retrospective single-center study of 97 patients with hematologic malignancies who underwent HSCT between August 2014 and May 2024. Patients received either HID transplantation (*n* = 57) or DUCB transplantation (*n* = 40). Hematopoietic recovery, graft-versus-host disease (GVHD), and long-term survival outcomes were assessed using cumulative incidence and Kaplan–Meier analyses, with multivariate Cox regression applied to identify prognostic factors.

**Results:**

HID recipients achieved significantly faster neutrophil (median: 14.00 vs. 22.50 days) and platelet recovery (median: 15.00 vs. 44.50 days) than DUCB recipients (both *P* < 0.001). The incidence of grade II–IV acute GVHD (16.80% vs. 47.70%, *P* < 0.001) and chronic GVHD (10.30% vs. 28.60%, *P* = 0.033) was lower in the HID group, although severe grade III–IV aGVHD rates were comparable (13.00% vs. 23.90%, *P* = 0.160). Five-year overall survival (56.70% HID vs. 48.90% DUCB, *P* = 0.573), relapse-free survival (76.60% vs. 89.70%, *P* = 0.210), GVHD-free relapse-free survival (48.00% vs. 46.30%, *P* = 0.410), and non-relapse mortality (NRM) (35.40% vs. 47.00%, *P* = 0.188) did not differ significantly between groups.

**Conclusion:**

Both HID and DUCB transplantation are viable alternatives in the absence of an HLA-matched sibling donor. However, HID transplantation offers faster hematopoietic recovery and a reduced GVHD burden, which may provide practical clinical advantages when donor availability permits.

## Introduction

Hematopoietic stem cell transplantation (HSCT) remains a cornerstone therapeutic modality for a wide spectrum of hematological malignancies, including leukemia, lymphoma, and myelodysplastic syndrome (MDS). Among available graft sources, a human leukocyte antigen (HLA)-matched sibling donor (MSD) is considered the gold standard, owing to its superior engraftment and immunologic compatibility. However, only approximately one-third of patients have access to an HLA-MSD, necessitating the exploration of alternative donor sources [1].

In the absence of a matched sibling, the two principal alternatives are haploidentical donors (HIDs) and umbilical cord blood (UCB) units [2]. HIDs, typically first-degree relatives, offer near-universal donor availability, making them especially valuable for individuals from underrepresented ethnic groups or with rare HLA haplotypes. Recent advances in transplant immunology—particularly the incorporation of post-transplant cyclophosphamide (PTCy)-based haploidentical transplantation and optimized donor-specific antibody management—have dramatically improved HID transplantation outcomes and broadened its clinical applicability [3]. Consequently, several large-scale studies have demonstrated that HID transplantation can achieve survival rates comparable to those of MSD transplantation [4–7].

UCB transplantation (UCBT), on the other hand, offers the advantage of requiring less stringent HLA matching [8], thus expanding donor accessibility for patients without an HLA-MSD [9]. Moreover, clinical outcomes of UCBT have been shown to approximate those of MSD transplantation, especially in younger populations [10–12]. Nevertheless, a significant limitation of UCBT lies in its low total nucleated cell (TNC) dose, which can delay hematopoietic recovery and increase the risk of transplant-related complications, particularly in adults. To address this, double-unit umbilical cord blood (DUCB) transplantation was developed, enabling an augmented cell dose and enhancing engraftment potential while maintaining immunological flexibility. Notably, DUCB transplantation has demonstrated survival outcomes on par with those of HID transplantation in selected patient cohorts [13].

As a landmark head-to-head comparative study, the BMT CTN 1101 trial [13] directly compared double-unit umbilical cord blood transplantation with HLA-haploidentical bone marrow transplantation and provided high-level evidence for alternative donor selection. Nevertheless, this trial focused on reduced-intensity conditioning and bone marrow graft sources, while real-world data regarding myeloablative, peripheral blood stem cell-based HID transplantation versus DUCB transplantation remain insufficient.

Despite substantial progress in both PTCy-haplo and DUCB transplantation platforms, persistent clinical uncertainty remains regarding the optimal choice between these two alternative donor strategies. While UCBT/DUCB is associated with a theoretically lower risk of GVHD, HID transplantation provides faster hematopoietic engraftment; these competing clinical benefits create a common dilemma in daily transplant practice. Direct, head-to-head comparisons assessing their relative efficacy in terms of engraftment kinetics, graft-versus-host disease (GVHD) incidence, and long-term survival remain limited. To address this gap, we conducted a retrospective single-center analysis aimed at systematically comparing survival outcomes, hematopoietic recovery, and transplant-related complications between DUCB and HID transplantation in patients with hematologic malignancies.

## Methods

### Study design

This retrospective, single-center study evaluated 97 patients diagnosed with hematologic malignancies who underwent allogeneic hematopoietic stem cell transplantation (allo-HSCT) at Xinhua Hospital, affiliated with Shanghai Jiao Tong University School of Medicine, between August 2014 and May 2024. All patients met established indications for transplantation. Patient allocation to either double-unit umbilical cord blood (DUCB) transplantation (*n* = 40) or haploidentical donor (HID) transplantation (*n* = 57) followed a predefined institutional donor selection algorithm.

For all enrolled patients without an HLA-matched related or unrelated donor, donor searches were initiated simultaneously for both haploidentical familial donors and umbilical cord blood units. Haploidentical hematopoietic stem cell transplantation (Haplo-HCT) was prioritized as the first-line option when a suitable familial haploidentical donor was available. DUCB transplantation was selected under the following conditions: (1) no eligible haploidentical donor was identified; (2) the presence of high-titer donor-specific antibodies (DSA), which may increase the risk of complications in the haploidentical transplant setting; and (3) availability of two umbilical cord blood units meeting predefined criteria, including a combined total nucleated cell (TNC) dose > 5.0 × 10⁷/kg recipient body weight and HLA compatibility of ≥ 4/6 matched loci. The final graft source allocation for each patient was therefore determined based on this hierarchical, availability-driven framework.

### Eligibility criteria

Patients were eligible for inclusion if they were between 10 and 70 years of age and had a confirmed diagnosis of a hematologic malignancy, including acute myeloid leukemia, acute lymphoblastic leukemia, myelodysplastic syndrome (MDS), high-grade lymphoma, or other rare hematologic disorders. All enrolled patients fulfilled established criteria for hematopoietic stem cell transplantation and received myeloablative conditioning prior to graft infusion. The study protocol was approved by the Institutional Review Board (IRB) of Xinhua Hospital, Shanghai Jiao Tong University School of Medicine (Approval Number: XHEC-D-2025-006). All procedures conformed to the ethical standards outlined in the 1975 Declaration of Helsinki. As this was a retrospective analysis, informed consent was waived.

### Procedure

High-resolution HLA typing for HLA-A, -B, -C, -DRB1, and -DQB1 loci was conducted for all donor–recipient pairs. A minimum of three out of six matched HLA loci was required to proceed with transplantation. In the HID group, HLA matching was assessed at high-resolution (allele-level) for HLA-A, -B, -C, and -DRB1 loci. In contrast, for DUCB transplantation, HLA matching was primarily based on antigen-level typing for HLA-A and -B, and allele-level typing for HLA-DRB1, consistent with standard cord blood transplantation practice.

In the HID group, peripheral blood stem cells were mobilized using granulocyte colony-stimulating factor (G-CSF) at a dose of 5 µg/kg/day for five consecutive days. For DUCB transplantation, each graft was required to provide a total nucleated cell (TNC) dose exceeding 2.5 × 10⁷ cells/kg. In cases involving two cord units, the combined TNC and CD34⁺ cell counts were used to assess adequacy [14]. Gender mismatch was defined as any sex disparity between the recipient and at least one donor unit. For DUCB recipients, the number of HLA matches was recorded based on the least matched cord unit.

### Conditioning regimen and graft-versus-host disease prophylaxis

All enrolled patients received a myeloablative conditioning protocol. The backbone conditioning regimen consisted of busulfan combined with cyclophosphamide and fludarabine in both the haploidentical donor (HID) transplantation group and the double-unit umbilical cord blood (DUCB) transplantation group, whereas the strategies for graft-versus-host disease (GVHD) prophylaxis differed significantly between the two cohorts. In the HID group, post-transplant cyclophosphamide (PTCy)-based GVHD prophylaxis was administered in combination with cyclosporine, as the initial calcineurin inhibitor (CNI) and mycophenolate mofetil. No anti-thymocyte globulin (ATG) was used in any patient undergoing HID transplantation in the present study. For patients in the DUCB group, the same myeloablative conditioning backbone (busulfan, cyclophosphamide, and fludarabine) was used. GVHD prophylaxis consisted of cyclosporine as the initial CNI and mycophenolate mofetil, while PTCy was not administered in this group.

### Endpoints and definitions

The primary study endpoint was overall survival (OS), defined as the duration from the date of transplantation to either the last follow-up or death from any cause. Secondary endpoints included relapse-free survival (RFS), hematopoietic recovery, graft-versus-host disease–free, relapse-free survival (GRFS), and non-relapse mortality (NRM).

To ensure inter-group comparability, relapse was diagnosed using unified, standardized criteria in both cohorts, as follows: Morphologic relapse: bone marrow blast proportion ≥ 5% on microscopic examination or pathologically/imaging-confirmed extramedullary leukemic infiltration. Molecular relapse: reappearance or elevation of disease-specific minimal residual disease (MRD) detected by flow cytometry or quantitative PCR, exceeding validated thresholds for each corresponding hematological malignancy. Clinical relapse: abnormal peripheral blood hematological parameters requiring urgent anti-tumor intervention in the absence of definite morphologic or molecular evidence.

Hematopoietic recovery was defined as achieving an absolute neutrophil count of ≥ 0.5 × 10⁹/L and a platelet count of ≥ 20 × 10⁹/L in the absence of transfusion support. Acute graft-versus-host disease (aGVHD) and chronic GVHD (cGVHD) were graded according to established criteria, including the modified Glucksberg grading system for aGVHD and the National Institutes of Health (NIH) consensus criteria for cGVHD [15, 16]. GRFS was defined as survival without the occurrence of grade III–IV aGVHD, severe cGVHD, disease relapse, or death.

### Statistical analysis

All statistical analyses were conducted using SPSS version 26.0 (IBM Corp., Armonk, NY, USA), GraphPad Prism version 8.0 (GraphPad Software, San Diego, CA, USA) and R software version 4.2.0. Categorical variables were compared using the chi-square (χ²) test, while continuous variables were assessed using independent-sample t-tests. Cumulative incidence functions were employed to estimate engraftment and GVHD rates, accounting for competing risks. Overall survival (OS), relapse-free survival (RFS), and GVHD-free, relapse-free survival (GRFS) were analyzed using the Kaplan–Meier method and compared using log-rank tests. Multivariate analysis was performed using the Cox proportional hazards regression model to identify independent prognostic factors. Cumulative incidence of NRM was calculated by treating relapse as a competing event, and differences between groups were compared using Gray’s test.

Given the inherent biological and logistical differences between the DUCB and HID cohorts—particularly regarding HLA matching grades and infused cell doses—propensity score matching (PSM) was explored but was not feasible due to substantial sample attrition and the intrinsic differences between graft sources, which would have compromised statistical power and generalizability. Consequently, we employed a multivariate Cox proportional hazards regression model to adjust for these potential confounders. Covariates were selected for inclusion based on the following criteria: (1) variables demonstrating a potential association with survival outcomes in univariate analysis (*P* < 0.10); and (2) variables of known clinical significance or those exhibiting substantial baseline imbalance between groups (including age, sex mismatch, HLA disparity, disease risk index, and infused cell dose), regardless of their univariate P-values. The donor type (DUCB vs. HID) was forced into the model as the primary variable of interest to evaluate its independent effect on survival outcomes. The variables retained in the final multivariate models are presented in Table 2. A two-sided p-value of < 0.05 was considered indicative of statistical significance.

## Results

### Patient characteristics

A total of 97 patients were included in this retrospective analysis, with 40 patients (41.20%) undergoing double-unit umbilical cord blood (DUCB) transplantation and 57 patients (58.80%) receiving haploidentical donor (HID) transplantation. The median follow-up time for the entire cohort was 77 months (interquartile range: 30.59-123.41 months). The median interval from initial diagnosis to transplantation was 209 days (range: 168–304) for the DUCB group and 186 days (range: 143–230) for the HID group. The median total nucleated cell (TNC) dose from donor grafts was 0.44 × 10⁸/kg (range: 0.37–0.55 × 10⁸/kg) in the DUCB cohort, compared with 14.12 × 10⁸/kg (range: 10.00–18.18 × 10⁸/kg) in the HID group. Similarly, the median CD34⁺ cell dose was 0.14 × 10⁶/kg (range: 0.09–0.20 × 10⁶/kg) in the DUCB group and 7.10 × 10⁶/kg (range: 3.49–8.95 × 10⁶/kg) in the HID group (Table 1). The baseline characteristics between the two cohorts were generally well-balanced, though some intergroup discrepancies were observed in variables including age, sex mismatch, and HLA matching status. These residual imbalances primarily stemmed from the inherent biological constraints associated with double-unit cord blood procurement.

**Table 1:** Patient, disease, and transplant characteristics according to group

	DUCB	HID	Total	*p* value
n(%)	40(41.24)	57(58.76)	(*n* = 97) (100%)	
Sex				0.359
Male	27 (67.50%)	32 (56.14%)	59 (60.82%)	
Female	13 (32.50%)	25 (43.86%)	38 (39.18%)	
Age at HSCT(years)				0.008
< 40	28 (70.00%)	23 (40.35%)	51 (52.58%)	
≥ 40	12 (30.00%)	34 (59.65%)	46 (47.42%)	
Diagnosis				0.195
ALL	20 (50.00%)	18 (31.58%)	38 (39.18%)	
AML	17 (42.50%)	30 (52.63%)	47 (48.45%)	
MDS	1 (2.50%)	6 (10.53%)	7 (7.22%)	
NHL&Other	2 (5.00%)	3 (5.26%)	5 (5.15%)	
Remission status				0.177
CR1	24 (60%)	41(71.93%)	65(67.00%)	
CR2	5 (12.50%)	3 (5.26%)	8 (8.25%)	
MDS	1 (2.50%)	6 (10.53%)	7 (7.22%)	
PD/NR	10 (25.00%)	7 (12.28%)	17 (17.53%)	
Refined disease risk index				0.422
Low	2 (5.00%)	1 (1.75%)	3 (3.09%)	
Intermediate	26 (65.00%)	45 (78.95%)	71 (73.20%)	
High	8 (20.00%)	8 (14.04%)	16 (16.49%)	
Very high	4 (10.00%)	3 (5.26%)	7 (7.22%)	
HCT-CI				0.703
0	33 (82.50%)	44 (77.19%)	77 (79.38%)	
≥ 1	7 (17.50%)	13 (22.81%)	20 (20.62%)	
Median days from diagnosis to HSCT	209.00 [168.00;304.00]	186.00 [143.00;230.00]	193.00 [148.00;267.00]	0.063
Sex.match				< 0.001
mismatch	32 (80.00%)	22 (38.60%)	54 (55.67%)	
match	8 (20.00%)	35 (61.40%)	43 (44.33%)	
ABO match, no.(%)				0.002
Matched	12 (30.00%)	34 (59.65%)	46 (47.42%)	
Major mismatched	16 (40.00%)	6 (10.53%)	22 (22.68%)	
Minor mismatched	9 (22.50%)	15 (26.32%)	24 (24.74%)	
Bidirectional mismatch	3 (7.50%)	2 (3.51%)	5 (5.15%)	
HLA match				< 0.001
3/6 − 4/6	22 (55.00%)	54 (94.74%)	76 (78.35%)	
5/6–6/6	18 (45.00%)	3 (5.26%)	21 (21.65%)	
TNC cells (×10^8^ /kg)	0.44 [0.37;0.55]	14.12 [10.00;18.18]	7.10 [0.45;16.00]	< 0.001
CD34^+^cells(×10^6^ /kg)	0.14 [0.09;0.20]	7.10 [3.49;8.95]	3.22 [0.15;7.94]	< 0.001

*DUCB* Double-unit umbilical cord blood, *HID* Haploidentical donors, *HSCT* Hematopoietic stem cell transplantation, *ALL* Acute lymphoblastic leukemia, *AML* Acute myeloid leukemia, *MDS* Myelodysplastic syndromes, *NHL* Non-Hodgkin’s lymphoma, *CR* Complete response, *PD* Progressive disease, *NR* Non-remission, *HCT-CI* hematopoietic cell transplantation-specific comorbidity index, *TNC* total nucleated cell

### Hematopoietic recovery

**Fig. 1:**
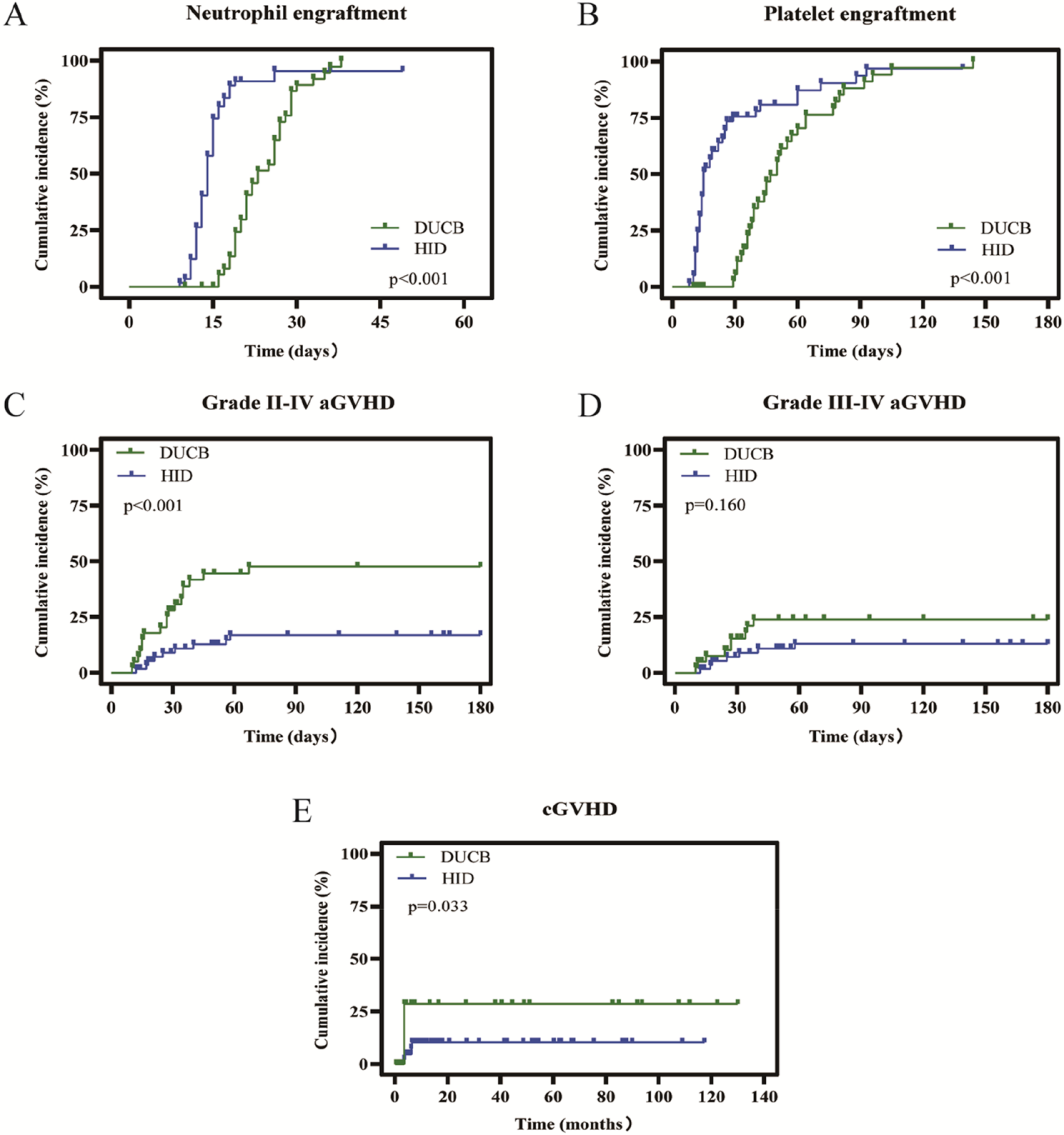
Hematopoietic recovery and incidence of graft-versus-host disease (GVHD) in DUCB and HID transplant recipients. **A** Cumulative incidence of neutrophil engraftment, demonstrating significantly faster recovery in the haploidentical donor (HID) group compared to the double-unit cord blood (DUCB) group (median: 14 vs. 22.5 days, *p* < 0.001). **B** Cumulative incidence of platelet engraftment, also significantly accelerated in HID recipients (median: 15 vs. 44.5 days, *p* < 0.001). **C** Incidence of grade II–IV acute GVHD (aGVHD), which was significantly lower in the HID group than in the DUCB group (16.8% vs. 47.7%, *p* < 0.001). **D** Incidence of grade III–IV aGVHD, which did not differ significantly between the groups (13% vs. 23.9%, *p* = 0.160). **E** Cumulative incidence of chronic GVHD (cGVHD), with a significantly higher rate observed in DUCB recipients compared to HID (28.6% vs. 10.3%, *p* = 0.033). DUCB = double-unit umbilical cord blood; HID = haploidentical donor; GVHD = graft-versus-host disease

The incidence of primary graft failure (non-engraftment) was comparable between the two groups, occurring in 8 of 57 patients (14.0%) in the HID group and 6 of 40 patients (15.0%) in the DUCB group (*P* = 0.89). Hematopoietic recovery was notably faster in patients receiving HID transplants compared to those who underwent DUCB transplantation. The median time to neutrophil engraftment was significantly shorter in the HID group (14.00 days) than in the DUCB group (22.50 days; *P* < 0.001). Similarly, platelet recovery occurred earlier in the HID cohort (15.00 days vs. 44.50 days; *P* < 0.001) (Fig. 1A and B). By day 28 post-transplant, neutrophil recovery had been achieved in 95.40% of HID recipients and 75.70% of DUCB recipients. At day 60, platelet recovery was observed in 87.20% of the HID group and 70.40% of the DUCB group. The prolonged neutropenic phase and delayed platelet engraftment in DUCB recipients may be associated with an elevated risk of bacterial, fungal, and viral infections, as well as increased transplant-related mortality (TRM) and non-relapse mortality (NRM) in the early post-transplant period. This early mortality burden may partially contribute to the comparable long-term survival observed between the two groups, highlighting the clinical impact of delayed hematopoietic reconstitution in DUCB transplantation.

### aGVHD and cGVHD

The cumulative incidence of grade II–IV acute graft-versus-host disease (aGVHD) by day 100 was significantly higher in the DUCB group compared to the HID group (47.70% vs. 16.80%; *p* < 0.001; Fig. 1C). In contrast, no statistically significant difference was observed in the incidence of severe grade III–IV aGVHD between the two cohorts (23.90% for DUCB vs. 13.00% for HID; *p* = 0.160; Fig. 1D). The 5-year cumulative incidence of chronic GVHD (cGVHD) was also elevated in the DUCB group relative to the HID group (28.60% vs. 10.30%; *p* = 0.033; Fig. 1E).

### OS, RFS, GRFS and NRM

**Fig. 2:**
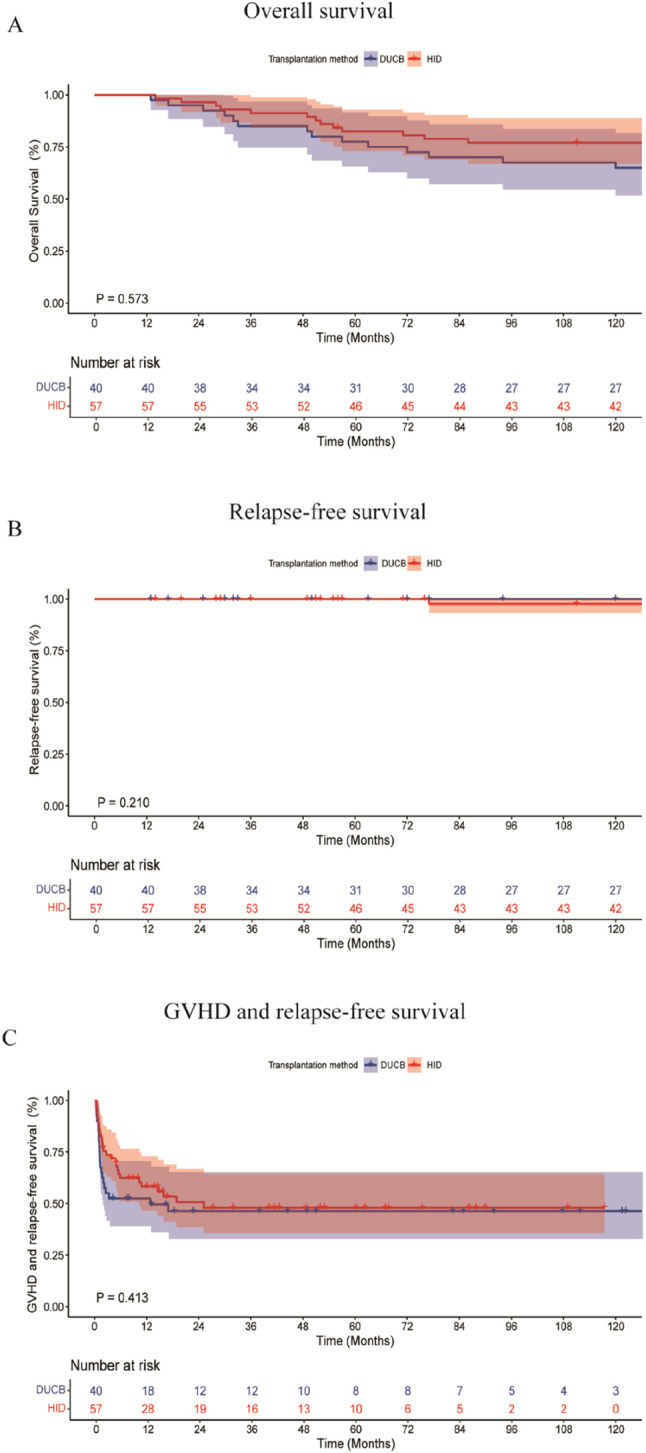
Long-term survival outcomes following double-unit umbilical cord blood (DUCB) and haploidentical donor (HID) transplantation. **A** Kaplan–Meier curve for overall survival (OS), showing no statistically significant difference between DUCB and HID groups (5-year OS: 48.9% vs. 56.7%, *p* = 0.573). **B** Relapse-free survival (RFS) was numerically higher in the DUCB group compared to HID (5-year RFS: 89.7% vs. 76.6%), but this difference did not reach statistical significance (*p* = 0.210). **C** GVHD-free, relapse-free survival (GRFS) was comparable between groups (5-year GRFS: 46.3% DUCB vs. 48.0% HID, *p* = 0.413). DUCB = double-unit umbilical cord blood; HID = haploidentical donor; OS = overall survival; RFS = relapse-free survival; GRFS = GVHD-free, relapse-free survival

**Fig. 3:**
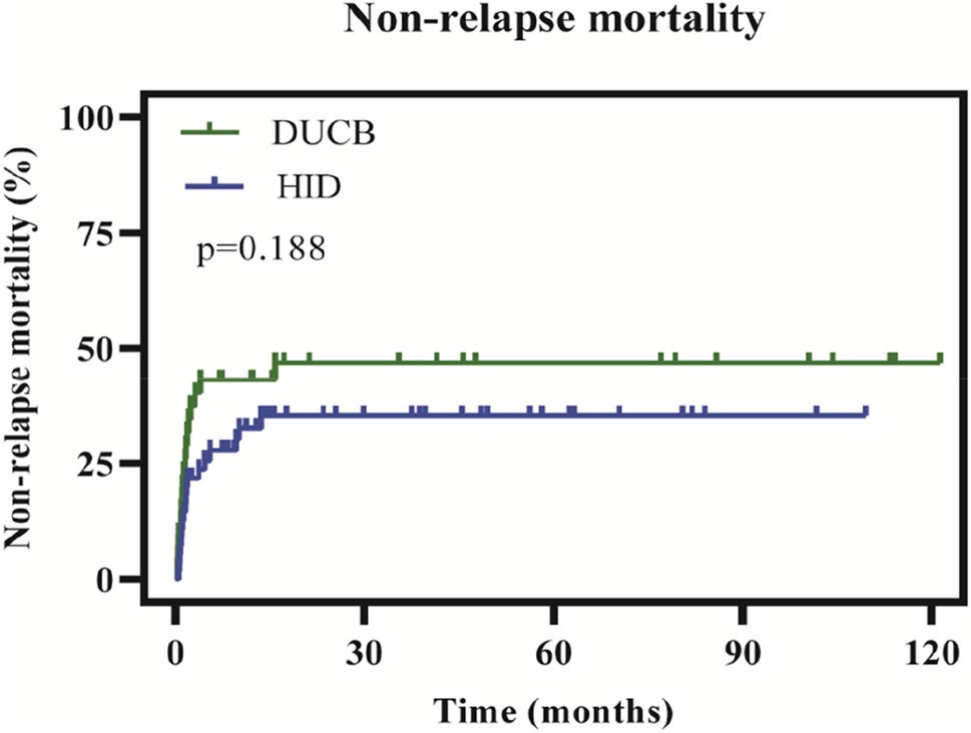
Cumulative incidence of non-relapse mortality (NRM) following double-unit umbilical cord blood (DUCB) and haploidentical donor (HID) transplantation. NRM was estimated using competing risk analysis, with relapse treated as a competing event. No statistically significant difference in NRM was observed between the DUCB and HID groups (*P* = 0.188). DUCB = double-unit umbilical cord blood; HID = haploidentical donor; NRM = non-relapse mortality

No significant difference in overall survival (OS) was observed between the two groups, with 5-year OS rates of 48.9% in the DUCB group and 56.70% in the HID group (HR 0.838, 95% CI 0.452–1.551, *P* = 0.573; Fig. 2A). Relapse-free survival (RFS) was numerically higher in the DUCB group (89.70%) compared to the HID group (76.60%), though this difference did not reach statistical significance (HR 2.329, 95% CI 0.621–8.735, *P* = 0.210; Fig. 2B). Although RFS was numerically higher in the DUCB group, this difference was not statistically significant and should be interpreted with caution, without drawing mechanistic conclusions. Similarly, the 5-year GVHD-free, relapse-free survival (GRFS) was comparable between groups, recorded at 46.30% for DUCB and 48.00% for HID (HR 0.784, 95% CI 0.437–1.405, *P* = 0.413; Fig. 2C). The cumulative incidence of non-relapse mortality (NRM), estimated using competing risk analysis, was 47.00% in the DUCB group and 35.40% in the HID group, with no statistically significant difference between the two groups (HR 0.631, 95% CI 0.318–1.251, *P* = 0.188; Fig. 3).

### Multivariate analysis of the main outcomes

Multivariate Cox regression analysis revealed no statistically significant differences in overall survival (OS), relapse-free survival (RFS), GVHD-free relapse-free survival (GRFS), or non-relapse mortality (NRM) between the DUCB and HID transplantation groups. However, several independent risk factors were identified. Patients with progressive disease or nonresponsive status at the time of transplantation exhibited significantly worse OS (hazard ratio [HR] = 3.182; 95% confidence interval [CI]: 1.565–6.471; *p* = 0.001). Similarly, a hematopoietic cell transplantation-specific comorbidity index (HCT-CI) ≥ 1 was associated with inferior OS (HR = 2.690; 95% CI: 1.363–5.310; *p* = 0.004), as was the presence of grade III–IV aGVHD (HR = 2.819; 95% CI: 1.377–5.770; *p* = 0.005). For RFS, gender mismatch emerged as a significant adverse factor (HR = 9.881; 95% CI: 1.235–79.060; *p* = 0.031). In the analysis of GRFS, both grade III–IV aGVHD (HR = 24.177; 95% CI: 10.525–55.541; *p* < 0.001) and HCT-CI ≥ 1 (HR = 3.095; 95% CI: 1.597–5.997; *p* = 0.001) were strongly associated with worse outcomes. These findings underscore the pivotal role of pre-transplant disease status, comorbidities, and severe GVHD in influencing long-term transplant success (Table 2).

**Table 2:** Multivariate analysis of survival and contributing factors

Outcomes	Hazard ratio (95% CI)	*P* value
*OS*		
Remission status		
CR1	reference	0.013
CR2	0.702 (0.163–3.025)	0.635
MDS	2.346 (0.787–6.997)	0.126
PD/NR	3.182 (1.565–6.471)	0.001
HCT-CI		
0	reference	
≥ 1	2.690 (1.363–5.310)	0.004
aGVHD		
0-II	reference	
III-IV	2.819 (1.377–5.770)	0.005
*RFS*		
Sex.match		
mismatch	reference	
match	9.881 (1.235–79.060)	0.031
*GRFS*		
HCT-CI		
0	reference	
≥ 1	3.095 (1.597–5.997)	0.001
aGVHD		
0-II	reference	
III-IV	24.177 (10.525–55.541)	< 0.001

*OS* overall survival, *RFS* relapse-free survival, *GRFS* GVHD-free, relapse-free survival, *CR* Complete response, *MDS* Myelodysplastic syndromes, *PD* Progressive disease, *NR* Non-remission, *HCT-CI* hematopoietic cell transplantation-specific comorbidity index, *aGVHD* acute graft-versus-host disease

## Discussion

Declining birth rates and aging populations have significantly reduced the availability of HLA-matched sibling donors, posing a critical barrier to allogeneic stem cell transplantation in patients with hematologic malignancies. Consequently, alternative graft sources such as umbilical cord blood (UCB), haploidentical donors (HIDs), and unrelated donors have become indispensable. However, the optimal graft choice among these alternatives remains a subject of ongoing debate [17]. The present study was designed to compare outcomes of HID and double-unit UCB (DUCB) transplantation following myeloablative conditioning in patients with hematologic malignancies.

In the present study, the HID group exhibited faster hematopoietic recovery and a relatively lower risk of severe GVHD compared with the DUCB cohort, which can be biologically explained. Our HID transplantation platform adopted a standardized PTCy-based prophylaxis strategy. Post-transplant cyclophosphamide selectively eliminates proliferating alloreactive conventional T cells while sparing and inducing the expansion of protective regulatory T cells (Tregs). The robust Treg expansion effectively inhibits excessive alloimmune responses, alleviates inflammatory tissue injury, and lowers the incidence and severity of GVHD. Moreover, PTCy-mediated immune modulation accelerates early lymphocyte and immune reconstitution, further facilitating rapid hematopoietic engraftment and stabilizing post-transplant hematopoietic function in haploidentical recipients. This immunological mechanism collectively accounts for the advantageous recovery and GVHD control observed in the HID transplantation group.

Our findings demonstrated that overall survival was comparable between the DUCB and HID groups. This observation is consistent with results from a multicenter, phase III clinical trial [13], which showed no significant difference in 2-year relapse-free survival (RFS) between DUCB and HID recipients undergoing reduced-intensity conditioning for leukemia and lymphoma. However, that study noted lower nonrelapse mortality and improved overall survival (OS) with haploidentical bone marrow transplantation. Similarly, a prospective trial comparing myeloablative single-unit UCB transplantation to HLA-haploidentical transplantation found no significant differences in 2-year OS, RFS, or nonrelapse mortality, though the UCB cohort had a lower 2-year GVHD-free, relapse-free survival (GRFS) [18]. Sugita et al. [19] also reported comparable 2-year OS and RFS between single-unit UCB and PTCy-haplo peripheral blood stem cell transplantation, with a significantly higher recurrence rate in the PTCy-haplo group.

Notably, the clinical relevance of our findings is particularly pronounced for transplant centers across Asia, where the practical landscape of alternative donor HSCT differs substantially from Western settings. Unlike North American and European cohorts, where matched unrelated donor (MUD) registries have extensive, population-wide coverage, Asian populations exhibit unique HLA haplotype diversity and strong regional specificity, resulting in significantly lower rates of fully matched unrelated donor availability for the majority of patients [20]. In this context, HID and DUCB have emerged as the two dominant, widely coexisting alternative donor sources in routine clinical practice across Asian countries, including China, Japan, and South Korea. Our study directly addresses a critical evidence gap for Asian clinical practice: the landmark BMT CTN 1101 trial was conducted primarily in Western populations, and focused on reduced-intensity conditioning and bone marrow grafts for HID transplantation, which does not reflect the mainstream clinical paradigm in Asia—where myeloablative conditioning and peripheral blood stem cell grafts with PTCy-based GVHD prophylaxis are the standard of care for HID transplantation. Furthermore, the clinical trade-offs between HID and DUCB in Asia are shaped by distinct regional factors: HID transplantation offers notable advantages in terms of lower procedural cost, simplified logistics without reliance on cord blood bank cold chain infrastructure, and near-universal immediate donor availability via first-degree relatives, which is particularly valuable in resource-limited Asian settings. In contrast, DUCB transplantation remains an indispensable option for patients without a suitable familial HID, those with high-titer donor-specific antibodies that preclude HID transplantation, or those requiring urgent transplantation without time for donor workup and mobilization, which aligns with real-world practice patterns reported in recent Asian multicenter studies [20]. Our 10-year head-to-head comparative data from a real-world Chinese (Asian) cohort therefore provide direct, practice-guiding evidence for stransplant centers in the region to balance these clinical, logistical, and economic factors when selecting the optimal alternative graft source for patients with hematologic malignancies.

In our cohort, the 5-year OS was 48.90% for DUCB and 56.70% for HID (*p* = 0.573); RFS was 89.70% versus 76.60% (*p* = 0.210), and GRFS was 46.30% versus 48.00% (*p* = 0.413), further supporting the clinical equivalency of both graft sources. However, hematopoietic recovery was significantly delayed in the DUCB group, as evidenced by prolonged neutrophil and platelet engraftment times. This delay may increase the risk of infection and bleeding, which are key contributors to early transplant-related mortality [13, 21, 22]. This delay prolongs the duration of severe neutropenia and thrombocytopenia, directly elevating the risk of severe opportunistic infections and life-threatening bleeding events. These complications are the primary drivers of increased TRM and NRM in the early post-transplant phase (within 100 days), leading to higher early mortality in DUCB recipients compared with HID recipients. Additional factors potentially contributing to the relatively high NRM in the DUCB cohort include lower HLA matching stringency compared with HID transplantation, which may increase alloreactivity and organ toxicity, as well as the absence of PTCy-mediated immune modulation that results in a higher incidence of grade II–IV acute GVHD and subsequent GVHD-related mortality. Furthermore, the inherently lower CD34⁺ cell dose in cord blood grafts impairs early immune reconstitution and further elevates infection susceptibility. Notably, the majority of NRM events in the DUCB group occurred within the first 6 months post-transplant, consistent with the temporal pattern of engraftment delay and early infectious or GVHD-related complications. Thus, delayed engraftment likely accounts for the elevated early mortality seen in UCB transplantation, even though long-term survival is comparable. Addressing this challenge remains a critical objective in optimizing DUCB outcomes, particularly through strategies to accelerate hematopoiesis and immune reconstitution to mitigate infection risk and reduce early NRM/TRM. UCB represents a valuable alternative graft source due to its immunologic tolerance and rapid availability and favorable immunologic characteristics. Numerous studies have reported encouraging long-term survival outcomes with UCB transplantation [10, 23, 24]. Nonetheless, its major limitation lies in the limited total nucleated cell (TNC) dose, which is frequently associated with delayed hematopoietic recovery and elevated transplant-related mortality [25, 26]. A TNC threshold of ≥ 2.5 0 × 10⁷/kg is considered essential for successful engraftment [27], a benchmark that can be difficult to meet in adult recipients or pediatric patients with higher body weights. DUCB transplantation offers a practical solution by combining two cord units to increase the overall cell dose, thus making it a valuable graft strategy [28].

Systematic reviews and meta-analyses have demonstrated that while single- and double-unit UCB transplantation produce comparable survival outcomes, DUCB transplantation may offer a lower risk of relapse at the cost of increased incidence of grade II–IV acute GVHD [26, 29]. In alignment with these findings, our study reported a significantly higher incidence of grade II–IV aGVHD in the DUCB group compared to the HID group (47.70% vs. 16.80%, *p* < 0.001), although grade III–IV aGVHD did not differ significantly between groups. Likewise, chronic GVHD was more frequent in DUCB recipients (28.60% vs. 10.30%, *p* = 0.033), consistent with observations reported by Sugita et al. [19]. The elevated aGVHD incidence in the DUCB group may be partially attributed to the use of myeloablative conditioning regimens [30]. In contrast, the post-transplant cyclophosphamide (PTCy) protocol employed in HID transplantation has been shown to effectively reduce the risk of both moderate and severe aGVHD [31]. While increased GVHD may theoretically enhance graft-versus-leukemia effects and reduce relapse risk, we did not observe a statistically significant difference in RFS between the two groups.

It is important to note that baseline characteristics such as HLA matching and cell dose differed between groups. These discrepancies are not indicative of selection bias but rather reflect the intrinsic biological constraints of double-unit cord blood procurement compared to haploidentical donation. Our multivariate analyses were specifically designed to adjust for these factors, ensuring that the comparative outcomes represent the effect of the graft type rather than baseline imbalances. This study has several limitations. Most notably, the relatively small sample size limited our ability to conduct subgroup analyses based on disease subtype, remission status, or patient age. Furthermore, we did not assess post-transplant immune reconstitution, which may play a pivotal role in determining GVHD incidence and transplant-related mortality. Future studies with larger cohorts and longer follow-up durations are warranted. In particular, evaluating immune recovery kinetics—such as lymphocyte subset reconstitution, T-cell receptor diversity, and cytokine profiling—may help clarify the mechanisms behind delayed immune reconstitution in DUCB recipients and guide the development of optimized transplantation protocols. Third, although we employed multivariate analysis to adjust for baseline imbalances, the relatively small sample size and limited number of events resulted in wide confidence intervals for some hazard ratios. This indicates uncertainty in the precise magnitude of the effect and suggests that the study may be underpowered to detect modest differences. Therefore, our findings regarding independent prognostic factors should be interpreted with caution and require validation in larger, multicenter cohorts. Lastly, although we recognize the importance of disease-specific outcomes, a subgroup analysis stratified by disease type (e.g., AML vs. ALL) was not feasible in this cohort due to limited sample size. Stratifying the patients into four groups (HID-AML, HID-ALL, DUCB-AML, DUCB-ALL) resulted in small cell counts that were underpowered for meaningful statistical comparison. Future multi-center studies with larger cohorts are needed to validate whether the observed survival benefits differ specifically between AML and ALL populations.

## Conclusion

Our single-center retrospective study over a 10-year real-world period provides head-to-head comparative data of myeloablative haploidentical donor (HID) versus double-unit umbilical cord blood (DUCB) transplantation for hematologic malignancies. We demonstrate that HID and DUCB transplantation achieve comparable long-term overall survival, relapse-free survival, and GVHD-free relapse-free survival, supporting both graft sources as feasible alternatives for patients without an HLA-matched sibling donor. While both approaches remain viable, HID transplantation may offer practical clinical advantages due to faster hematopoietic recovery and a lower incidence of grade II–IV acute and chronic GVHD. These benefits may translate into a reduced risk of early transplant-related complications, including infection and bleeding, and an improved peri-transplant safety profile. DUCB transplantation remains an important alternative for patients without access to a suitable haploidentical donor, particularly in settings where donor availability or immunological constraints limit HID use. However, strategies to enhance immune reconstitution and accelerate engraftment are needed to further improve DUCB outcomes, especially in adult populations. Importantly, our findings do not demonstrate superiority of HID over DUCB in terms of long-term survival outcomes. Therefore, these results should be interpreted with caution, considering the retrospective design, limited sample size, and baseline imbalances between cohorts. Further large-scale, prospective studies are warranted to validate these findings and optimize graft selection strategies.

### Limitation of study

This study has several limitations. First, the retrospective, single-center design may introduce selection bias and limit generalizability. Second, the relatively small sample size restricted the ability to perform subgroup analyses by disease subtype, age, or remission status. Third, immune reconstitution data were not assessed, preventing evaluation of post-transplant immune recovery and its correlation with GVHD and infection risk. Although non-relapse mortality (NRM) was evaluated using competing risk analysis, detailed assessment of cause-specific mortality and temporal patterns (early versus late NRM) was not performed, which may limit further interpretation of transplant-related risks. Additionally, long-term follow-up data on late complications such as secondary malignancies or organ toxicity were not available. Future multicenter, prospective studies with larger cohorts and immune monitoring are warranted to validate and expand upon these findings.

### Future prospectives

Future research should focus on large-scale, multicenter prospective studies to validate the comparative outcomes of DUCB and HID transplantation. Emphasis should be placed on evaluating post-transplant immune reconstitution, including lymphocyte subset recovery, T-cell receptor diversity, and cytokine profiles. Such analyses could clarify mechanisms underlying delayed hematopoiesis and higher GVHD rates in DUCB recipients. Additionally, refining conditioning regimens and graft manipulation strategies may help enhance engraftment and reduce complications. Incorporating long-term follow-up to assess late effects, quality of life, and relapse patterns will be essential for optimizing graft source selection and improving overall transplant success.

## Data Availability

The datasets generated and analyzed during the current study are not publicly available due to institutional confidentiality policies but are available from the corresponding author upon reasonable request.

## Abbreviations

HSCT: Hematopoietic stem cell transplantation
HID: Haploidentical donor
DUCB: Double-unit umbilical cord blood
HLA: Human leukocyte antigen
MSD: Matched sibling donor
PTCy: Post-transplant cyclophosphamide
UCB: Umbilical cord blood
TNC: Total nucleated cell
GVHD: Graft-versus-host disease
OS: Overall survival
RFS: Relapse-free survival
GRFS: GVHD-free relapse-free survival
NRM: Non-relapse mortality
TRM: Transplant-related mortality
IRB: Institutional Review Board
G-CSF: Granulocyte colony-stimulating factor
DSA: Donor-specific antibody
ATG: Anti-thymocyte globulin
MRD: Minimal residual disease
NIH: National Institutes of Health
PSM: Propensity score matching
HCT-CI: Hematopoietic cell transplantation-specific comorbidity index
CI: Confidence interval
HR: Hazard ratio
